# Pigmented epithelioid melanocytoma (PEM) of the spine with compression fracture: case report

**DOI:** 10.1186/s12891-021-04923-0

**Published:** 2022-01-03

**Authors:** Sarthak Nepal, Borriwat Santipas, Wasan Yotchai, Manasmon Chairatchaneeboon, Sirichai Wilartratsami, Panya Luksanapruksa

**Affiliations:** 1 Division of Spine Surgery, Department of Orthopedic Surgery, Faculty of Medicine Siriraj Hospital, Mahidol University, 2 Wanglang Road, Bangkok Noi, Bangkok, 10700 Thailand; 2 Department of Pathology, Faculty of Medicine Siriraj Hospital, Mahidol University, Bangkok, Thailand; 3 Department of Dermatology, Faculty of Medicine Siriraj Hospital, Mahidol University, Bangkok, Thailand

**Keywords:** Pigmented epithelioid melanocytoma, PEM, Spine, Compression fracture, Case report

## Abstract

**Background:**

Pigmented epithelioid melanocytoma (PEM) is a sporadic type of pigmented melanocytic tumor with uncertain malignant potential. PEM arises as a solitary neoplasm that predominantly occurs spontaneously in otherwise healthy patients. Due to its rarity, a gold standard treatment regimen does not exist; however, symptomatic cases should be managed with radiotherapy and surgery.

**Case presentation:**

A 28-year-old Thai female presented with a sudden onset of back pain and weakness of the lower extremities during the postpartum period. Magnetic resonance imaging demonstrated abnormal soft tissue formation from T4 to T7; it extended to the vertebral bodies, left neural foramina, and posterior columns of T6 and T7. The patient underwent complete tumor debulking, decompressive laminectomy from T4 to T8, and posterior instrumentation from T3 to T10. The histopathology and immunohistochemistry suggested PEM. The patient fully resolved back pain after surgery. Nevertheless, as the patient re-presented with a neurological deficit a few months after the operative intervention, it was decided to perform a surgical resection via an en bloc vertebrectomy. At the one-year follow-up, although the patient reported continued improvement of her back pain, there was no motor power improvement.

**Conclusions:**

Spinal cord compression due to PEM is uncommon, especially in adults. Early diagnosis and treatment provide a good prognosis and help to regain lost neurological functions. Complete tumor removal and decompression of the spinal cord must be considered as a treatment strategy. Perioperative radiotherapy and chemotherapy have also been highlighted as treatment modalities for spinal tumors. With our reported case, early operative intervention coupled with radiotherapy produced satisfying outcomes.

## Background

Pigmented epithelioid melanocytoma is a sporadic, pigmented melanocytic tumor having an uncertain malignant potential. The term “pigmented epithelioid melanocytoma” (PEM) was coined by Zembowicz et al. in 2004. The morphological spectrum of the condition comprises animal-type melanoma and low-grade epithelioid blue nevus of the carney complex [1]. Although PEM and cellular blue nevi have similar architectural features, the presence of pigmented and clear epithelioid cells in PEM differentiates it from blue nevi.

PEM most commonly occurs in children, adolescents, and young adults. The most common occurrence site is the extremities (40.3%) [2]; the other sites are the head and neck region, the trunk, and the genitalia. Metastasis in PEM rarely occurs compared with other melanocytic tumors. Studies have reported PEM metastasizing to lymph nodes and rarely to distant sites (liver, spleen, bone marrow, and parotid glands) [3, 4]. The present study describes a case of PEM of the T6 and T7 vertebrae with a severe compression fracture causing an incomplete spinal cord injury a day after normal vaginal delivery.

## Case presentation

A 28-year-old Thai female (G3P1A1) without any previous medical illness presented with sudden-onset severe pain in the upper back and weakness of her lower limbs. The previous day, she had been admitted to the hospital for normal vaginal delivery. Immediately after the birth, the patient developed sudden-onset upper back pain. Within a few hours of delivery, she developed progressive weakness of her legs and urinary incontinence. On examination, she had a visible kyphotic deformity around the upper thoracic spine and marked tenderness on palpation at the midthoracic level. A neurological examination revealed the motor power of her legs to be grade II/V on all of the right side and grade I/V on the left side, except at L2, which showed grade II/V. The pinprick sensation was impaired below T7 level in both legs. Although the patient also had a loose sphincter tone, there was the presence of both anal wink and deep anal pressure. No clonus was detected. Based on the American Spinal Injury Association (ASIA) impairment scale, she was classified as having an incomplete spinal cord injury (ASIA B).

**Fig. 1:**
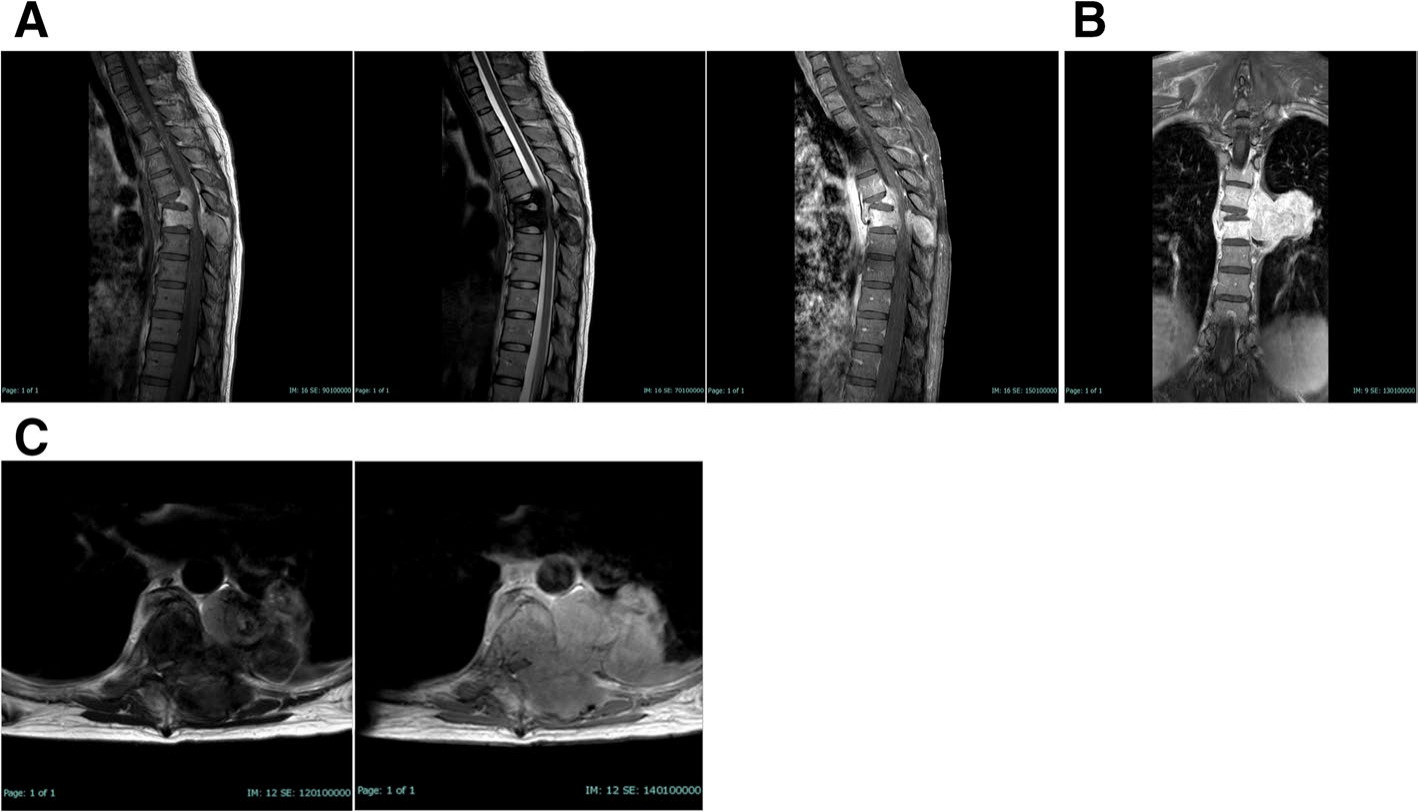
MR-image of thoracic spine showed abnormal bone marrow signal intensity at T6 and T7 vertebral levels which show high signal intensity (SI) on T1W, low SI on T2W and STIR with enhancement (**A**). In the axial cut, adjacent left paravertebral soft tissue formation at lower border of T4-T7 levels which show heterogeneous high SI on T1W, heterogeneous low signal on T2W with intense enhancement involving vertebral body (**B**). Left neural foramen, posterior column of T7 level, and left posterior 7th ribs, causing severe spinal canal stenosis and spinal cord compression at T5 - T7 levels (**C**)

Magnetic resonance imaging (MRI) of the entire spine revealed abnormal bone marrow signal intensity at T6 and T7, with adjacent paravertebral soft tissue formation from the T4 to the T7 levels. The lesion appeared with heterogeneous high-signal intensity on T1WI but with heterogenous low-signal intensity on T2WI/STIR. There was also an enhancement involving the vertebral bodies, left neural foramina, and posterior columns of T6 and T7. Additionally, a severe compression fracture at the T6 level and a mild compression fracture at the T7 level were observed. Furthermore, multiple nodules scattered throughout both lungs were visible, suggestive of lung metastases. The imaging findings are described in Fig. 1.

**Fig. 2:**
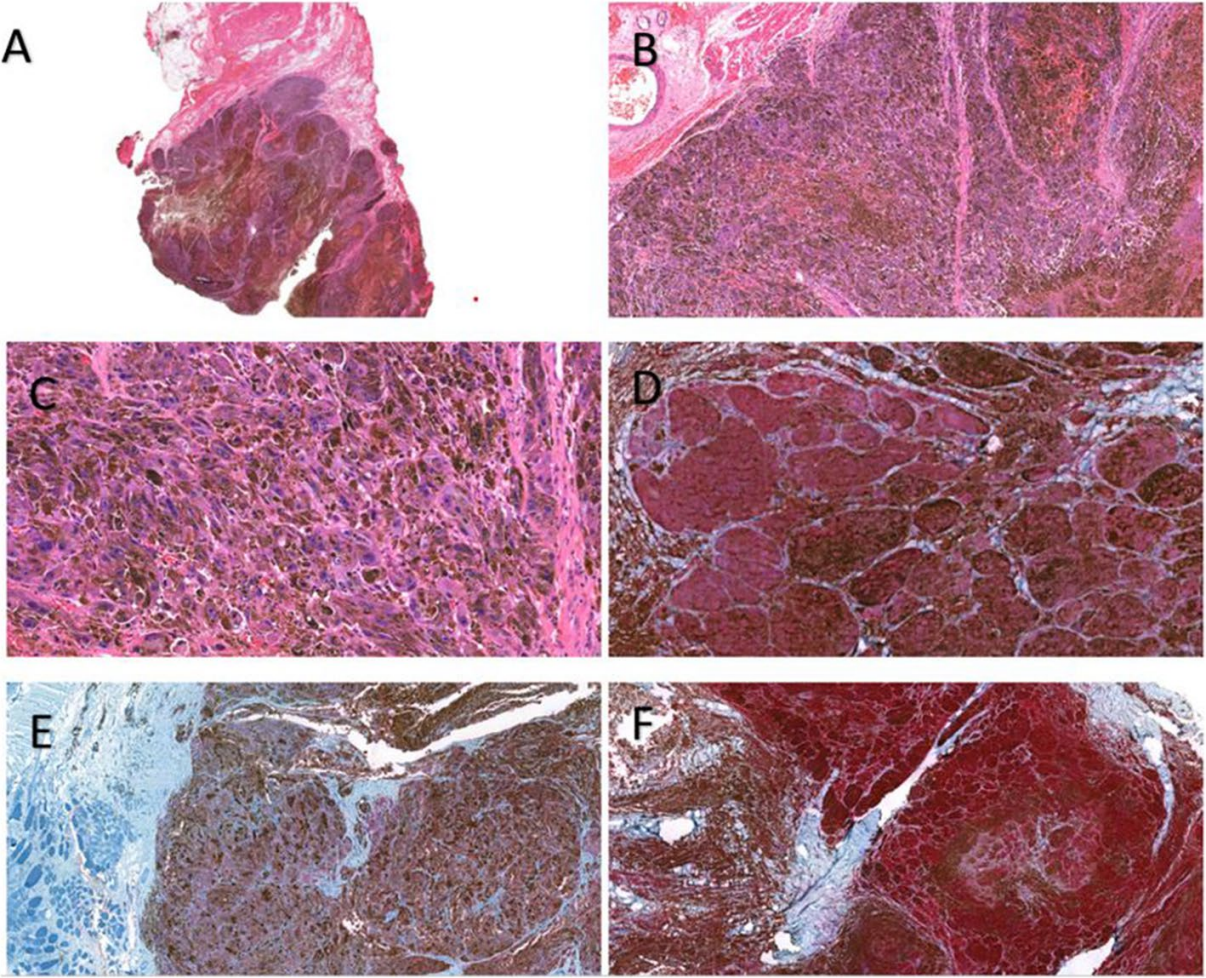
**A** and **B** The low magnification shows a heavily pigmented tumor infiltrating into the surrounding skeletal muscle fibers (H&E, A:40×, B: 100×); **C** the tumor is composed of predominantly medium to large-sized epithelioid cells with heavily pigmented cytoplasm obscuring nuclei, some of them have large vesicular nuclei with prominent nucleoli and abundant cytoplasm, Spindled blue nevus-like cells constitute the minor component, rare mitotic activity are seen (H&E, 400×); **D** Tumor cells were positive for S-100 protein (Red Chromogen: 200×); **E** Tumor cells were positive for MART-1/MELAN-A (Red Chromogen: 200×); **F** Tumor cells were positive HMB-45 (Red Chromogen: 200×)

**Fig. 3:**
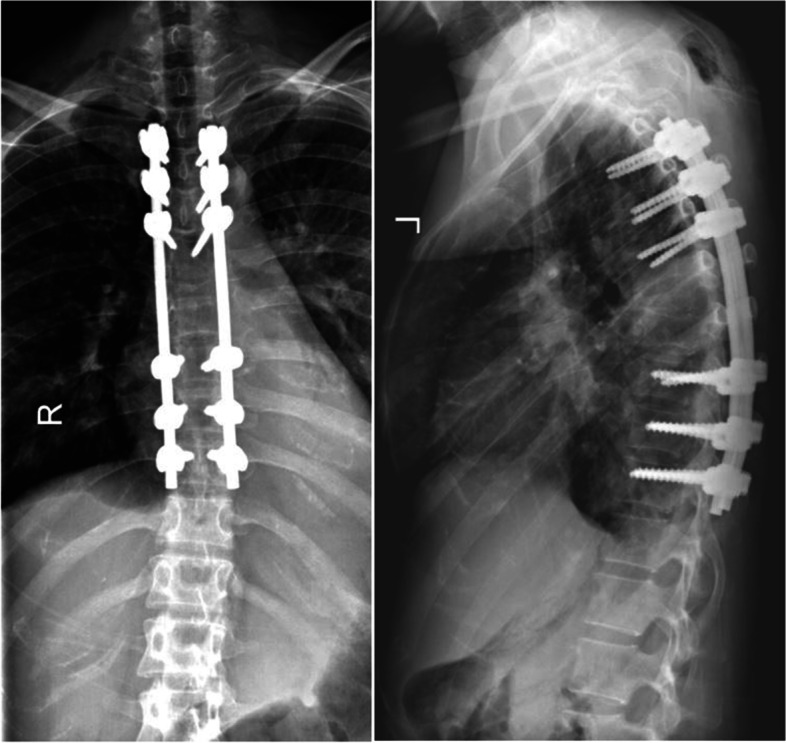
Post-operative radiograph showed the satify instrument position and spinal alignment

**Fig. 4:**
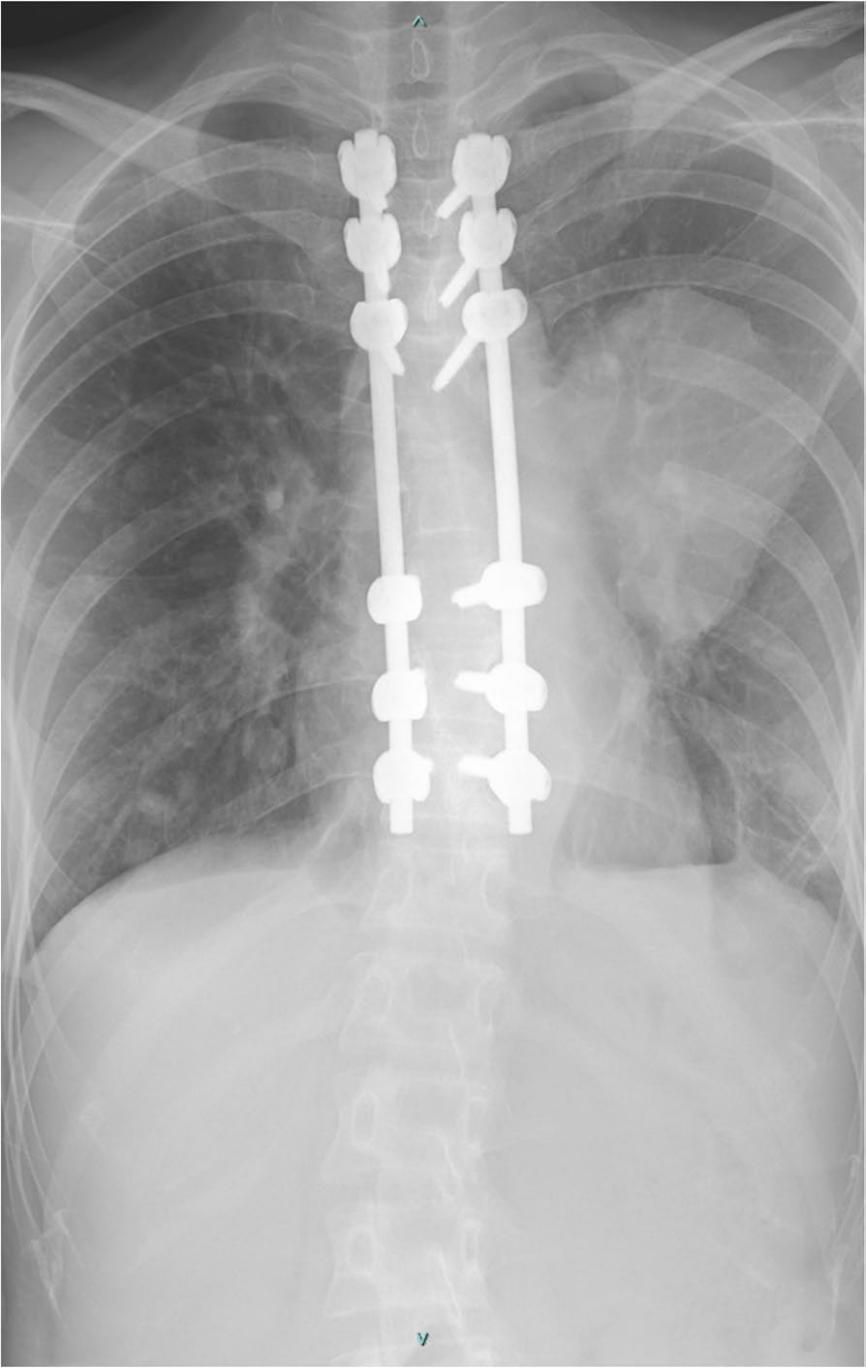
Post-operative radiograph of second operation

She was brought to operating room within 6 h after the occurrence of symptoms. Subperiosteal paraspinal muscle dissection was performed, after which partial tumor debulking at the T6 and T7 levels was done. Pedicular screws were then inserted from the T3 to the T10 levels—other than at the T6 and T7 levels—with the aid of the O-arm navigation system. Subsequently, complete tumor debulking was undertaken at the T6 and T7 levels. The tumor appeared as a sloppy mass containing a greenish-black secretion. A decompressive laminectomy was performed between the T4 and T8 levels, and posterior instrumentation was done between the T3 and T10 levels. Histopathological analysis of tissue samples from the T6 and T7 levels revealed heavily pigmented melanocytic lesions. They were composed of medium-to-large epithelioid cells with large vesicular nuclei, prominent macronuclei, and abundant cytoplasmic melanin pigment. The tumor demonstrated an infiltrative growth pattern at the periphery. Mitotic activity was relatively low (0–1/high power field). Psammoma bodies and adipose-like cells were not observed. Tumor necrosis averaged 20% of the tumor volume. A surrounding soft-tissue invasion and bone invasion were evident. The histopathological details are described in Fig. 2. Immunohistochemically, the tumor was positive for melanin, vimentin, S100 protein, human melanoma black (HMB)-45, and Melan A. Staining for epithelial membrane antigen (EMA) was immunoreactive. However, iron and CD34 were negative. The absence of the features of psammoma bodies, adipose-like cells, EWSR1 rearrangement, nuclear anaplasia, and brisk mitotic activity meant that melanocytic schwannoma, clear cell sarcoma, and malignant melanoma were excluded. Consolidation of the various findings led to a diagnosis suggestive of PEM of the spine. Postoperatively, the patient was consulted by an oncologist and given radiotherapy. Post-operative radiographs were shown in Figs. 3 and 4.

### Outcomes and follow-up

Immediately postoperatively, the patient’s back pain was subsided, and improvement was seen in her neurological status, with an assessment of grade C on the ASIA impairment scale. Nonetheless, upon a neurological assessment during 2 months postoperative follow-up, the motor power of both legs was grades 0/V, with grade B on the ASIA impairment scale. The pinprick sensation below the T4 level had also decreased on the right side and was absent on the left. Both clonus and the Babinski reflex were present bilaterally. The patient underwent a second operation 2 months after the first. The fourth to ninth ribs resection and resection of the residual tumor was done. Histopathological analysis revealed a residual PEM. Postoperatively, the patient’s neurological status reverted to grade C on the ASIA impairment scale. At the one-year follow-up, although the patient reported continued improvement of her back pain, there was no motor power improvement.

## Discussion and conclusion

Melanocytic tumors of the central nervous system (CNS) are exceedingly rare, solitary, low-grade, pigmented neoplasms, and they may arise anywhere along the neural axis. The term “melanocytoma” was first used in 1972 to report a heavily pigmented tumor of the foramen magnum [5]. These tumors are slow-growing and are locally aggressively, with spinal locations being rare.

PEMs fit into the nosological category of borderline melanocytic tumors. No gender predilection has been observed, and sun exposure is not involved in the pathogenesis of PEM [6]. The predominant causes of PEM involve a loss in expression of the Carney complex gene, cyclic adenosine 3′,5′ monophosphate-dependent protein kinase regulatory subunit 1alpha (Prkar1α). There have been no reports of PEMs being found in the spines of adults.

### Etiology

Melanocytic lesions of the nervous system and its covering are considered to originate from leptomeningeal melanocytes, which are derivatives of the neural crest during the embryonic stage of development. Accordingly, melanocytic lesions of the central nervous system were classified by Brat et al. [7] as low grade (melanocytoma), intermediate grade, and high grade, based on the cytological features focal mass lesion. Subsequently, primary melanocytic lesions were classified by the World Health Organization as diffuse melanocytosis, melanocytoma, malignant melanoma, and meningeal melanocytosis [8]. Lesions can be located in the ventral medulla, high cervical area, or—uncommonly—thoracolumbar spinal cord.

Diffuse melanocytosis and melanomatosis involve leptomeninges and might invade the parenchyma. They can present with signs of malignancy. Primary leptomeningeal melanomatosis is an aggressive tumor that carries a poor prognosis [9]. Meningeal melanocytoma consists of solitary, benign, low-grade tumors. They occur commonly in the extramedullary intradural compartment of the cervical and thoracic spines [10]. Primary melanomas of the central nervous system are aggressive in nature but account for only 1% of all melanoma cases. Leptomeningeal melanoma presents with signs of malignancy [7]. A close relationship between these tumors and the spinal nerve root is often noted.

The incidence of pregnancy-related spinal tumors is about 1 in 1000 to 2000 pregnancies [11]. They are usually diagnosed in the third trimester or within a year of the postpartum period. Several theories about the cause of the growth of these spinal tumors have been put forward. A few have suggested that the elevation of estrogen and progesterone levels during pregnancy can result in the development of these tumors. Additionally, increase growth factors, angiogenic factors, an increase in intravascular volume, biochemical changes, and hemodynamics have been suggested as playing a part in expanding the tumor size, resulting in spinal compression [12, 13].

### Clinical presentation

The atypical clinical presentations, radiographic features, and distinct histological features of specific types of spinal tumors aid in making a definitive diagnosis.

Primary and metastatic tumors of the spine are often asymptomatic or have nonspecific symptoms. The symptoms depend on the location and size of the tumor, and the degree of compression of the spinal cord or nerve root. If symptomatic, pain is a crucial presenting feature; it might result from mechanical instability of the vertebral column or neural compression [14]. Red-flag signs of severe thoracic pain, night pain, and pain that is “gnawing” in nature can be present. Spinal instability and neurological compromise are critical symptoms that might occur in these patients [15].

Plain radiography reveals indirect signs of a mass effect. A chest x-ray could be obtained as a part of the initial work-up. A computed tomography scan of the chest, abdomen, and pelvis could be used to stage the disease, identify metastases and lymph node involvement, and facilitate surgical planning [9]. MRI is very effective in assessing local spread and staging. The pigmented tumor of the spine shows a high-signal intensity on a T1-weighted image but a low-signal intensity on a T2-weighted image [16]. However, one disadvantage of MRI is that it sometimes overestimates the extent of the tissue involvement.

The histopathological features suggestive of PEM are a cellular element proliferation with epithelioid cytoplasm containing melanin pigment infiltrating the papillary dermis in blocks. In immunohistochemistry studies, PEM demonstrates positivity for Melan A, HMB-45, and S-100 [17, 18]. PEM has been described as behaving less aggressively than conventional melanomas. However, our study contrasts with the other reported findings as our patient experienced an aggressive disease course, with further metastasis being detected in the lungs.

### Treatments

The appropriate management of spinal tumors is debatable. Surgery and radiotherapy can be used simultaneously for the treatment of spinal tumors. Perioperative radiotherapy and brachytherapy have been extensively employed as management measures. However, both the optimal radiotherapy dosage and the method to combine surgery with radiotherapy to obtain the best outcomes are still being debated. Rades et al. [19] suggested using high-dose local radiation to prevent recurrence even when complete resection of the tumor is achieved. In their study, a 100%, 5-year, local-disease control was achieved when complete resections and radiotherapy were used together as the modality of treatment. This outcome was markedly higher than the 80%, 5-year, local-disease control achieved when only a complete resection was done. Like the published management strategy, our case also received a complete resection of the tumor along with radiation.

Local administration of chemotherapy could be an option for eradication of residual tumor cells after excision. Systemic chemotherapy is not used for spinal tumors because of the potential for side effects caused by the treatment strategy. Local administration of paclitaxel has shown efficacious therapeutic effects; it additionally has a radio-sensitizing property that aids local tumor control. However, chemotherapy or other therapeutic strategies has not led to benefit in the case of PEM [20].

If a tumor occurs in the spinal region, the surgical goal is to completely resect the tumor and decompress the spinal cord. The main surgical procedures are percutaneous vertebroplasty, debulking surgery, piecemeal resection, and total en bloc spondylectomy. Tumor related nerve compression can be relieved by using piecemeal resection and debulking surgery. Total en bloc spondylectomy involves removing an entire vertebral body and posterior elements to achieve tumor resection with negative margins. Evidence from the literature also indicates that gross total resection is another treatment option for central nervous system tumors [21]. There is evidence of favourable outcomes in patients undergoing complete resection of mass in PEM. Hence, gross-total surgical resection is seen as the standard of care for the treatment of PEM [20].

Long-term clinical follow-up of a patient is necessary after surgical resection and adjuvant therapy to monitor the recurrence of the tumor and determine the outcomes. With PEM, the published long-term follow-up studies do not report any adverse outcomes in cases with metastasis to the sentinel lymph nodes [22]. Metastasis to the lymph nodes does not suggest a malignant clinical course in PEM. However, our case demonstrated malignant behavior as there was metastasis to the lungs. Therefore, the prognosis in our case is not as favourable as mentioned in the literature for PEM.

In summary, this case highlights a rare case of PEM of the spine causing incomplete cord injury from a compression fracture.

PEM is an extremely rare form of pigmented melanocytic tumor. It has an uncertain malignant potential, and it predominantly occurs spontaneously in healthy patients. The rarity of the tumor creates difficulties in establishing a diagnosis. MRI is the modality of choice for patients with suspicion of spinal cord compression resulting from the tumor. Complete tumor removal and decompression of the spinal cord must be considered as part of the treatment strategy. Perioperative radiotherapy and chemotherapy have also been highlighted as modalities of treatment for spinal tumors. With our reported case, early operative intervention combined with radiotherapy produced satisfying outcomes.

## Data Availability

The datasets generated and analyzed during the current study are available from the corresponding author on reasonable request.

## Abbreviations

PEM: Pigmented epithelioid melanocytoma
ASIA: American Spinal Injury Association
MRI: Magnetic resonance imaging
CNS: Central nervous system
EMA: Epithelial membrane antigen
